# Ethnic Differences in Neonatal Body Composition in a Multi-Ethnic Population and the Impact of Parental Factors: A Population-Based Cohort Study

**DOI:** 10.1371/journal.pone.0073058

**Published:** 2013-08-29

**Authors:** Line Sletner, Britt Nakstad, Chittaranjan S. Yajnik, Kjersti Mørkrid, Siri Vangen, Mari H. Vårdal, Ingar M. Holme, Kåre I. Birkeland, Anne Karen Jenum

**Affiliations:** 1 Institute of Clinical Medicine, University of Oslo, Oslo, Norway; 2 Department of Child and Adolescents Medicine, Akershus University Hospital, Lørenskog, Norway; 3 Norwegian Resource Centre for Women's Health, Oslo University Hospital, Oslo, Norway; 4 Diabetes Unit, King Edward Memorial Hospital, Pune, India; 5 Department of Endocrinology, Oslo University Hospital, Oslo, Norway; 6 Unit of Biostatistics and Epidemiology, Oslo University Hospital, Oslo, Norway; 7 Department of General Practice, Institute of Health and Society, University of Oslo, Oslo, Norway; 8 Faculty of Health, Oslo and Akershus University College of Applied Sciences, Oslo, Norway; Iran University of Medical Sciences, Iran (Republic of Islamic)

## Abstract

**Background:**

Neonates from low and middle income countries (LAMIC) tend to have lower birth weight compared with Western European (WE) neonates. Parental height, BMI and maternal parity, age and educational level often differ according to ethnic background, and are associated with offspring birth weight. Less is known about how these factors affect ethnic differences in neonatal body composition.

**Objectives:**

To explore differences in neonatal body composition in a multi-ethnic population, and the impact of key parental factors on these differences.

**Methods:**

A population-based cohort study of pregnant mothers, fathers and their offspring, living in Oslo, Norway. Gender- and gestational-specific z-scores were calculated for several anthropometric measurements, with the neonates of WE ethnic origin as reference. Mean z-scores for neonates with LAMIC origin, and their parents, are presented as outcome variables.

**Results:**

537 singleton, term neonates and their parents were included. All anthropometric measurements were smaller in neonates with LAMIC origin. Abdominal circumference and ponderal index differed the most from WE (mean z-score: −0.57 (95% CI:−0.69 to −0.44) and −0.54 (−0.66 to −0.44), and remained so after adjusting for parental size. Head circumference and skin folds differed less, and length the least (−0.21 (−0.35 to −0.07)). These measures became comparable to WEs when adjusted for parental factors.

**Conclusions:**

LAMIC origin neonates were relatively “thin-fat”, as indicated by reduced AC and ponderal index and relatively preserved length and skin folds, compared with neonates with WE origin. This phenotype may predispose to type 2 diabetes.

## Introduction

There is now strong evidence supporting that early life environment plays a powerful role in influencing later susceptibility to chronic diseases [1]. This involves mechanisms of developmental plasticity, including epigenetic processes [2]. These adaptive mechanisms enable the development of an offspring with a phenotype appropriate for the environment in which it is predicted to live. The associations between low birth weight (BW), and also high BW, and later risk of cardiovascular disease (CVD) and type 2 diabetes are reported in several populations [3]–[6]. However, subtle variations in environmental influence, such as maternal size, metabolism and nutrition can probably produce a range of neonatal phenotypes which may affect the risk of adult disease, even in the absence of large effects on birth weight [4]–[7].

The prevalence of type 2 diabetes and CVD is particularly high in many Asian populations [8], and also in ethnic minority groups living in high income countries [9], [10]. The smallest neonates are observed in South Asia. These neonates have been shown to have a “thin-fat phenotype”, defined as small abdominal viscera and low muscle mass, but preserved body fat compared with Europeans [11]–[13]. In Europe and North America most ethnic minority groups originating from low and middle income countries (LAMIC), have lower mean BW than ethnic Europeans. The lowest BW is observed in South Asian ethnic minorities, independent of whether the mother was born in South Asia or in the high income country of residence, whereas mean BW in ethnic Middle Eastern neonates often is similar to that of ethnic Europeans [14]–[17].

Offspring size is determined by several factors. Parental size, age, parity and socioeconomic and nutritional conditions are known to influence fetal growth and birth weight [18]. Strong associations between parental factors and offspring anthropometrics are observed in several populations living in their original context [19]–[22]. However, less is known about these relations and the impact of parental factors on ethnic differences in neonatal body composition in multi-ethnic European populations.

The aim of this study was to assess ethnic differences in neonatal body composition in offspring of women with expected normal pregnancies, comparing neonates with ethnic origin from LAMIC with those of Western European (WE) origin. Secondly, we wanted to examine if parental size, parity, age and educational level could explain these differences.

## Methods

### Ethics statement

The women were given oral and written information about the Stork Groruddalen project when attending the CHC for antenatal care and invited to participate. The women who chose to participate gave informed written consent at inclusion, on behalf of themselves and their offspring. The fathers were given written information after inclusion of the mother and gave separate informed written consent. The study protocol and the consent-forms were approved by The Regional Committee for Medical and Health Research Ethics for South Eastern Norway, and The Norwegian Data Inspectorate.

### Population and design

This population-based cohort study was set up at three public Health Clinics in Oslo, Norway, covering districts with an ethnic- and socioeconomic diverse population [23]. Antenatal care in normal pregnancies is provided in primary care, and the public health clinics are attended by the majority (75–85%) of pregnant women in this area.

The study methods, including maternal anthropometric measurements are presented in detail elsewhere [23]. Information material and questionnaires were translated to eight languages: Arabic, English, Sorani, Somali, Tamile, Turkish, Urdu, and Vietnamese, covering the largest ethnic groups. They were eligible if they were: (1) living in one of the districts, (2) would give birth at the study hospitals, (3) were in gestational week <20, (4) not suffering from diseases necessitating intensive hospital follow-up during pregnancy (i.e. pre-gestational diabetes and other substantial medical, psychiatric or obstetrical conditions) (5) not already included with a pregnancy lasting >22 weeks, (6) could communicate in Norwegian or any of the other eight languages, and (7) were able to give informed consent. The inclusion period was from May 2008 to May 2011. Of those eligible overall participation rate was 74%. The study cohort of 823 women was representative for the main ethnic groups, and there were no significant ethnic differences in proportions excluded by different criteria [23], [24].

### Parental factors

Maternal questionnaire data (by interview) and anthropometric measurements were collected at inclusion [23]. Paternal questionnaire data included self-reported height, weight and ethnicity. Body height was measured to the nearest 0.1 cm using a fixed stadiometer and body weight to the nearest 0.1 kg with an electronic scale. Skin folds were measured twice at three sites (triceps, subscapular and suprailiac, with Holtain T/W Caliper 0–48 mm (Holtain Ltd., Crymych; UK), and the mean of these two is used. Sum of skin folds are the sum of measurements at all three sites. Parity was classified as primiparous or one or more previous viable pregnancies. In Norway dating of pregnancy in usually based on routine ultrasound measurements performed in gestational week (GW) 17–20. However, the assumption that the growth rate of all fetuses is similar until this time may not be true. Further, for comparison of our results with similar studies, gestational week (GW) was derived from the first day of the woman's last menstrual period (LMP). Term was calculated as date of LMP +282 days. In 37 (7%) LMP date was unknown/uncertain, or differed >14 days from ultrasound term, or there was an IVF-pregnancy. Ultrasound term (from routine scan) was then used in calculations of GW. Extensive data on medical and obstetric history were collected retrospectively from hospital medical birth records for all mother/neonate pairs, and all dates relevant to calculating term were double-checked.

We defined ethnic origin by the participant's country of birth or the participant's mother's country of birth, if the participant's mother was born outside Europe or North-America [23]. Women with ethnic origin from Asia, Middle East, Africa and South-/Central-America were categorized as LAMIC women according to World Bank classification of countries 2008. Women with ethnic origin from WE (91% Norwegian born) and North America were categorized as WE women (reference group). Women born in Eastern Europe were handled separately, and excluded from the main analyses, as they were few and have experienced a different socioeconomic context than WE immigrants. The women were further categorized into eight ethnic subgroups (WE, Eastern Europe, Pakistan, Sri Lanka/India, East Asia, Middle East, Sub-Sahara Africa and a small heterogeneous group from South/Central America) taking both geographical and cultural factors into account (Supplementary table 1) [23], [25].

**Table 1:** Characteristics of mothers, pregnancies, neonates and fathers. Data presented as mean (SD) or n (%).

	Western European^a^	LAMIC^b^
	n = 229	n = 282
**Mothers**		
Age at inclusion, years	30.7 (4.5)	29.1 (5.0)
Primipara (%)	118 (52)	105 (37)
Educational level		
Primary school or less (<10 years) (%)	7 (3)	77 (28)
High school (10–12 years) (%)	76 (33)	132 (47)
College/university education (%)	145 (64)	71 (25)
Norwegian born (%)	209 (91)	31 (11)
Gestational weeks at inclusion.	14.2 (2.2)	15.7 (4.0)
Height, cm	167.5 (5.7)	160.6 (6.0)
Weight, kg, at inclusion	70.7 (13.2)	65.1 (14.4)
BMI, kg/m^2^, at inclusion	25.2 (4.5)	25.2 (5.0)
Sum of skin folds, mm	70.3 (19.5)	73.5 (20.3)
**Pregnancies/births**		
Previous stillbirth (% of multiparous)	1 (1)	4 (2)
Previous spontaneous abortion (%)	32 (14)	63 (22)
Previous cesarean section (% of multiparous)	16 (14)	34 (19)
Smoking at inclusion (daily or occationally) (%)	12 (5)	4 (1)
Mild hypertension (HT)/pre-eclampsia (%)	12 (5)	8 (3)
Severe HT/pre-eclampsia/eclampsia/HELLP-s.^c^ (%)	1 (0.4)	4 (1)
Gestational diabetes (WHO-criteria) (%)	23 (10)	37 (14)
Spontaneous start of labour (%)	177 (77)	225 (80)
Birth complications d (%)	66 (29)	97 (35)
**Neonates**		
Gestational age, days e	283 (9)	279 (9)
Gender, boy (%)	119 (52)	137 (49)
Apgar <8, 1 min	22 (10)	18 (6)
SGA, <10 perc (%)^f^	19 (8)	63 (22)
LGA, >90 perc (%)^f^	19 (8)	11 (4)
Birth weight, g	3600 (467)	3326 (476)
**Study-specific measurements**		
CH-length, cm^g^	50.0 (1.9)	49.4 (1.9)
Placenta weight, g ^h^	696 (142)	656 (148)
Head circ., cm	35.1 (1.3)	34.6 (1.4)
Abdominal circ. (umbilicus), cm	32.7 (2.2)	31.4 (2.1)
Sum of skin folds, mm	70.3 (19.5)	73.5 (20.3)
Ponderal index, kg/m^3^	28.7 (2.4)	27.5 (2.5)
**Fathers with complete data (n = 414)**	n = 209 (91)	n = 189
Ethnicity same as mother (%)	190 (92)	173 (93)
Height, cm	181.1 (6.3)	175.1 (7.6)
Weight, kg	87.0 (13.4)	80.7 (14.0)
BMI, kg/m^2^	26.5 (3.8)	26.3 (4.0)

a Western Europe (n = 229, 7 from other Scandinavian countries than Norway, 5 with other Western-European background (3 born in North America)).

b Women with ethnic origin from low- and middle-income countries in Asia, Middle East, Africa and south/central-America.

(includes two women from East Asian countries now classified as high income countries).

c HELLP: severe complication to preeclampsia (HEmolysis, ELevated liver enzymes and Low Platelet count).

d Composite of four birth complications; meconium-stained amniotic fluid, Apgar <7 after 5 min, grade 3–4 perineal tear or acute caesarean section.

e Based on last menstrual period for all births in study sample, includes 37 with ultrasound-derived term.

f Small for gestational age (SGA) and large for gestational age (LGA), calculated from Norwegian national references, stratified by GW and sex.

g Missing in 43 (8%) neonates, mostly due to intrauterine breech position, family history of hip-dysplasia or other circumstances restricting stretching of the baby.

### Measurements of neonatal body composition

**Figure 1:**
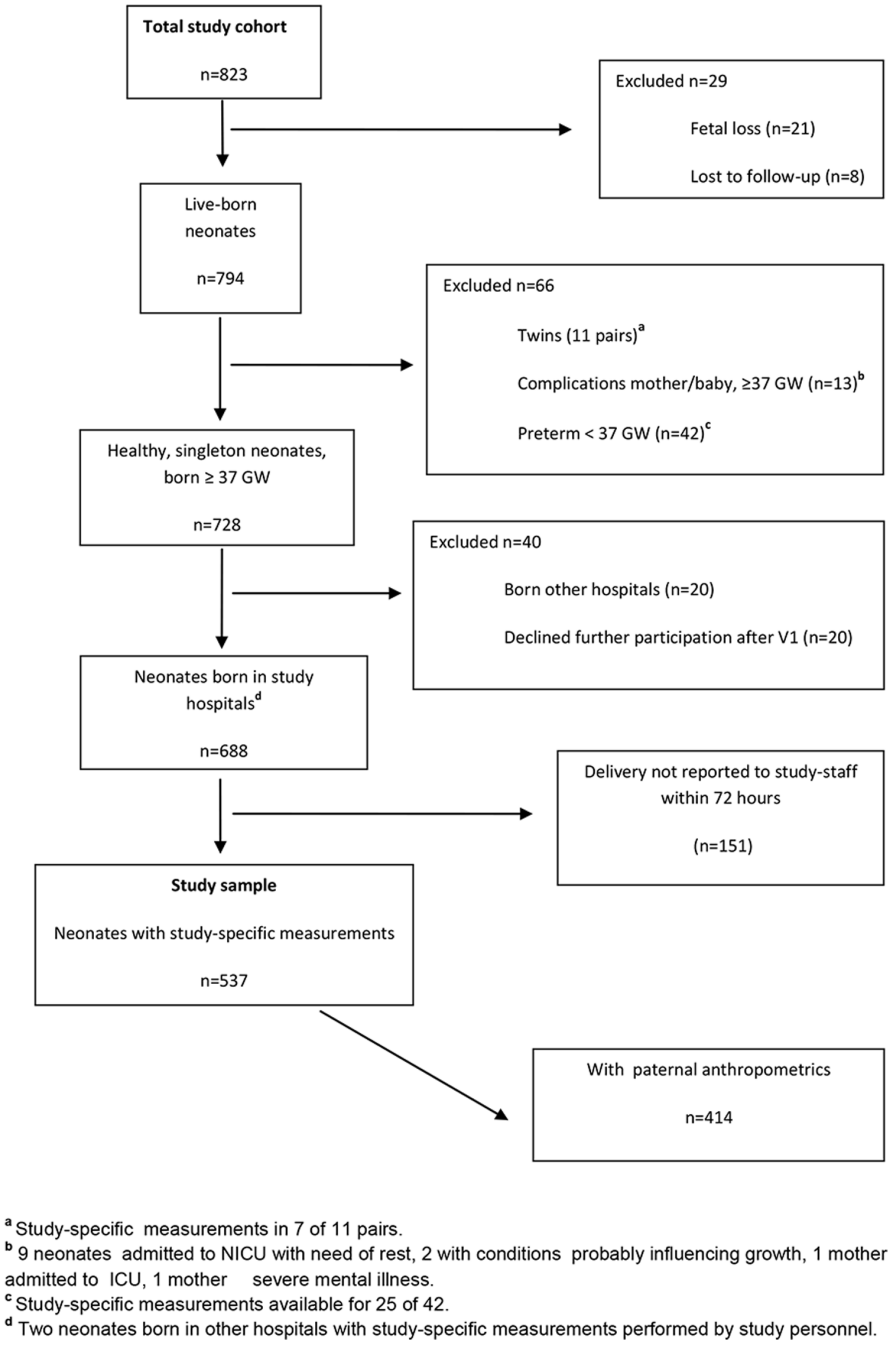
Flow diagram showing parent-neonate-pairs selected for analysis.

BW and placenta weight (PW), including cord and membranes, were routinely measured on electronic scales, calibrated by study staff (maximum difference: 5 g), immediately after birth. Within 72 hours after birth study-specific anthropometric measurements were performed by specially trained study personnel, unless contraindicated because of medical conditions restricting handling of the neonate (Figure 1).

Crown-heel length (CH-length) was measured to the nearest 0.1 cm, by a measuring rod, with the head firmly held, while stretching the legs. For circumference measurements a non-elastic plastic tape was used. Skin fold thickness was measured with a similar caliper as for maternal measurements. Abdominal and chest circumferences (AC) were measured at the umbilical and processus xiphoideus (sternum) levels, mid-upper arm circumference (MUAC) and triceps skin fold at the mid-point between the acromion and the elbow and thigh circumference and skin fold between hip and knee, at front, at the maximum circumference. Sub-scapular skin fold was measured below the lower angle of the scapula and the supra-iliac skin fold at the mid-point between the lowest rib and the iliac crest. Head circumference (HC) was measured with the tape tightened just above the eyebrows and the largest protuberance of the skull. Other circumferences were measured without compressing the underlying tissue. All measurements, except length, were performed twice (circumferences to the nearest 0.1 cm, skin folds to the nearest 0.1 mm), and the means were used. A third measurement was performed if a difference >0.5 cm for circumferences and >0.5 mm for skin folds, using the mean of two measurements with a difference <0.5 cm/mm. Sum of skin folds represents the sum of all four skin fold measurements. Ponderal index is BW (kg)/CH-length (m^3^). The two study-midwives measured 75%, specially trained stand-ins 25% of the neonates.

### Statistical methods

Anthropometric measurements were normally distributed. Differences between the study sample and those without measurements were tested by independent sample t-tests for continuous data and chi square tests for categorical data, with a significance level of 5%. With the WE group used as reference, individual z-scores were calculated (z-score = Observation – WE mean)/WE SD). For neonatal anthropometrics z-score-calculations were stratified by GW (37, 38, 39, 40 and 41+) and gender. Hence, z-scores indicate the number of standard deviations an observation is above or below the mean of the reference population. To validate our data, we computed z-scores for BW using the latest available national data (all births in Norway 1967–1998, mainly ethnic Norwegians) as reference [26]. A mean z-score of 0.01 was found for our WE reference group, indicating a representative sample.

Inter-rater variability, expressed as % Technical Error of Measurement (%TEM) [27], was assessed biannually. Inter-rater variability ranged from 5 to 21% between study personnel regarding maternal skin folds, and from 0.9% and 1.8% regarding neonatal CH-length and circumferences, to 8–13% for neonatal skin folds. Intra-rater variability was less than 5% in all measurements for all study-midwifes.

Z-scores of PW, BW, CH-length, HC, ponderal index, AC and sum of skin folds were chosen as dependant variables representing different aspects of growth, using general linear models (GLM) for each of these outcomes. Estimated marginal means for the outcome variables by ethnic groups were extracted, unadjusted (Model 1), adjusted for maternal anthropometry and parity (Model 2; primipara vs. para 1+, maternal z-scores of height and BMI), and with additional adjustments for maternal age and education (three categories; primary school or less, high school or college/university education) (Model 3). In the sub-sample of neonates with paternal data, additional adjustments for paternal height and BMI were performed (Model 4). One-Way-Anova with Bonferroni correction was used to test for heterogeneity within the LAMIC-groups. All analyses were done using SPSS 19, except for the reliability-analyses, where the statistical programming language R 2.11.0 was used.

## Results

### Characteristics of study sample

A total of 823 women were enrolled (Figure 1). All neonates were eligible for study-specific measurements by study-personnel unless contraindicated for medical reasons (Figure 1). Among both WE and LAMIC neonates, 6% were preterm (<37 GW) and excluded from analyses along with 11 twin-pairs, while 728 participants delivered a “healthy”, singleton term baby (GW 37+). Study-specific measurements were obtained from 537 neonates, constituting the study sample. (Figure 1) A sub-sample of 414 also had paternal data.

Eleven percent of LAMIC women (n = 31, mostly of Pakistani origin) in the study sample were born in Norway or another high-income country. LAMIC women were slightly younger and fewer were primiparous or had college/university education than WE women (all p<0.05) (Table 1). The pregnancies of LAMIC women were on average three days shorter. No large differences in pregnancy characteristics were found between WE and LAMIC women (Table 1). The study sample was comparable to the 191 healthy, term, singleton babies without study-specific anthropometric measurements for all factors listed in Table 1 (p-value ranging from 0.4–0.9), also when stratified by ethnicity.

### Body composition and impact of parental size and parity

**Figure 2:**
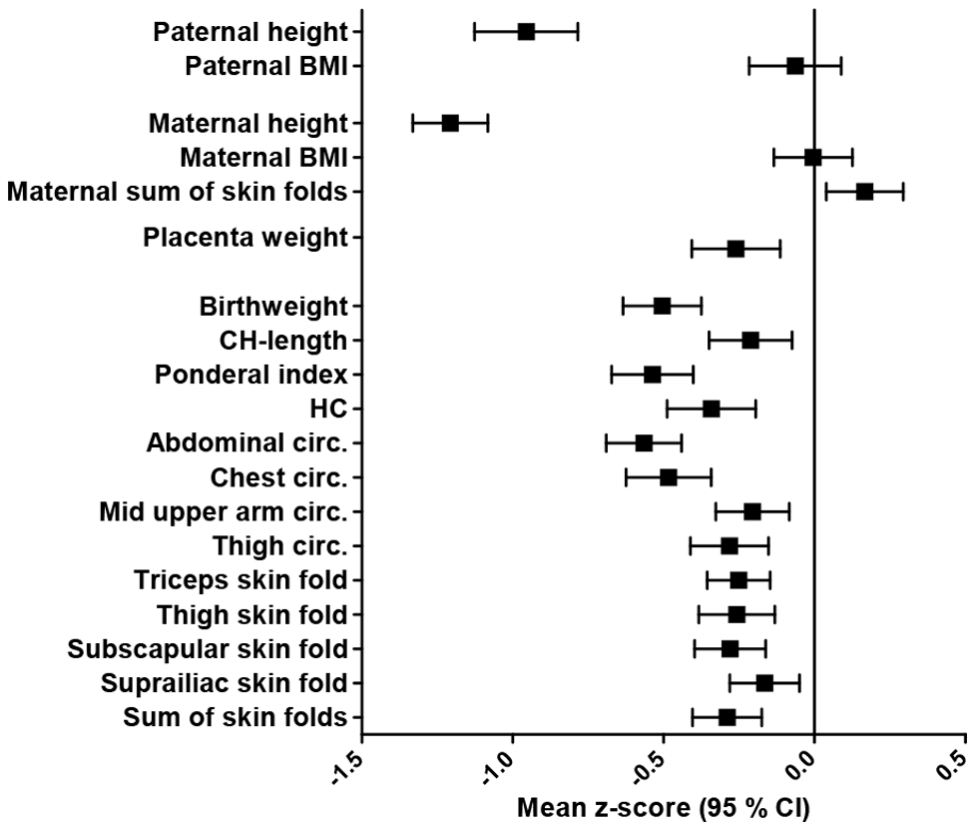
Crude mean z-scores (95% CI) of anthropometric measurements, for parents, placentas and neonates with ethnic origin from LAMIC, with Western Europeans as reference.

LAMIC women were substantially shorter with a mean z-score (sd) of −1.21 (−1.33, −1.08), had a similar BMI, and larger sum of skin folds than WEs (0.17 (0.04, 0.29) (Figure 2). The pattern was similar for paternal anthropometrics. All neonatal measurements were significantly smaller in the LAMIC group (Figure 2).

**Figure 3:**
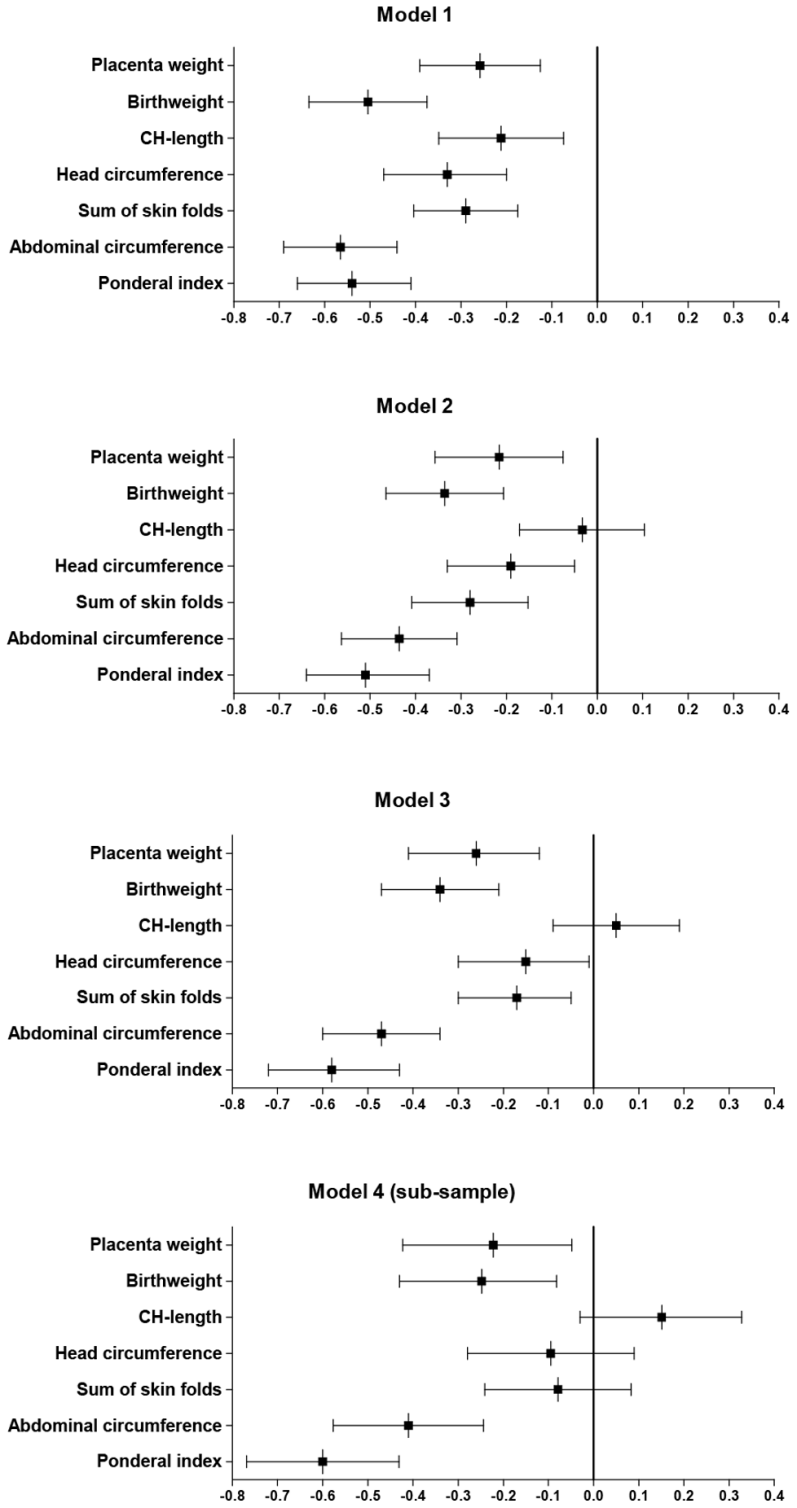
Mean z-scores (95% CI) for selected anthropometric measurements for neonates with ethnic origin from LAMIC with ethnic Western Europeans as reference. Model 1: unadjusted estimates. Model 2: estimates adjusted for maternal parity, height and BMI. Model 3: estimates adjusted for maternal factors as in model 2 and maternal age and education. Model 4: estimates adjusted for all factors as in model 3 and paternal height and BMI.

Figure 3 presents the mean z-scores for selected measurements for LAMIC neonates. In the unadjusted model (Model 1) BW, ponderal index and AC were most reduced in LAMIC neonates with a mean neonatal z-score of −0.50 (−0.63, −0.37), −0.54 (−0.66, −0.41) and −0.57 (−0.69, −0.44), respectively, compared with WE. Sum of skin folds and HC were less and CH-length least affected (−0.21 (−0.35, −0.07), p = 0.03).

When adjusting for maternal anthropometry and parity (Model 2), differences between LAMIC and WE neonates were reduced for all measurements (mean adjusted z-score for BW: −0.34 (−0.47, −0.21)), except for ponderal index (−0.51 (−0.64, −0.37)). Additional adjustments for maternal education and age (Model 3) further reduced differences for HC, CH-length and skin folds, but not for BW, AC and ponderal index (−0.58 (−0.72, −0.47). Thus, the thin-fat phenotype became more evident.

With additional adjustments for paternal factors in the sub-sample (n = 414), ethnic differences persisted for ponderal index (−0.60 −0.7, −0.43) and were still highly significant (p<0.001) for AC. Finally, when repeating the analyses in Model 1, 2 and 3 for those with complete paternal data, no changes in the estimates of effect size were observed, although wider confidence intervals were observed due to loss of power.

### Parental-neonatal relationships

Parity was positively associated with all neonatal measurements, weakest with PW (p = 0.014). Maternal and paternal height were positively associated with all measurements except with PW and sum of skin folds, and maternal BMI with all except CH-length. No associations were found between paternal BMI and all neonatal measurements, and maternal age was only associated with sum of skin folds. When analysing the impact of maternal educational level, a positive trend was observed in bivariate analyses. However, significant differences were only observed between the lowest and the highest category. No significant interactions were found between maternal factors when explored in models with adjustments for maternal education and age (Model 3).

### Heterogeneity across ethnic minority sub-groups

**Figure 4:**
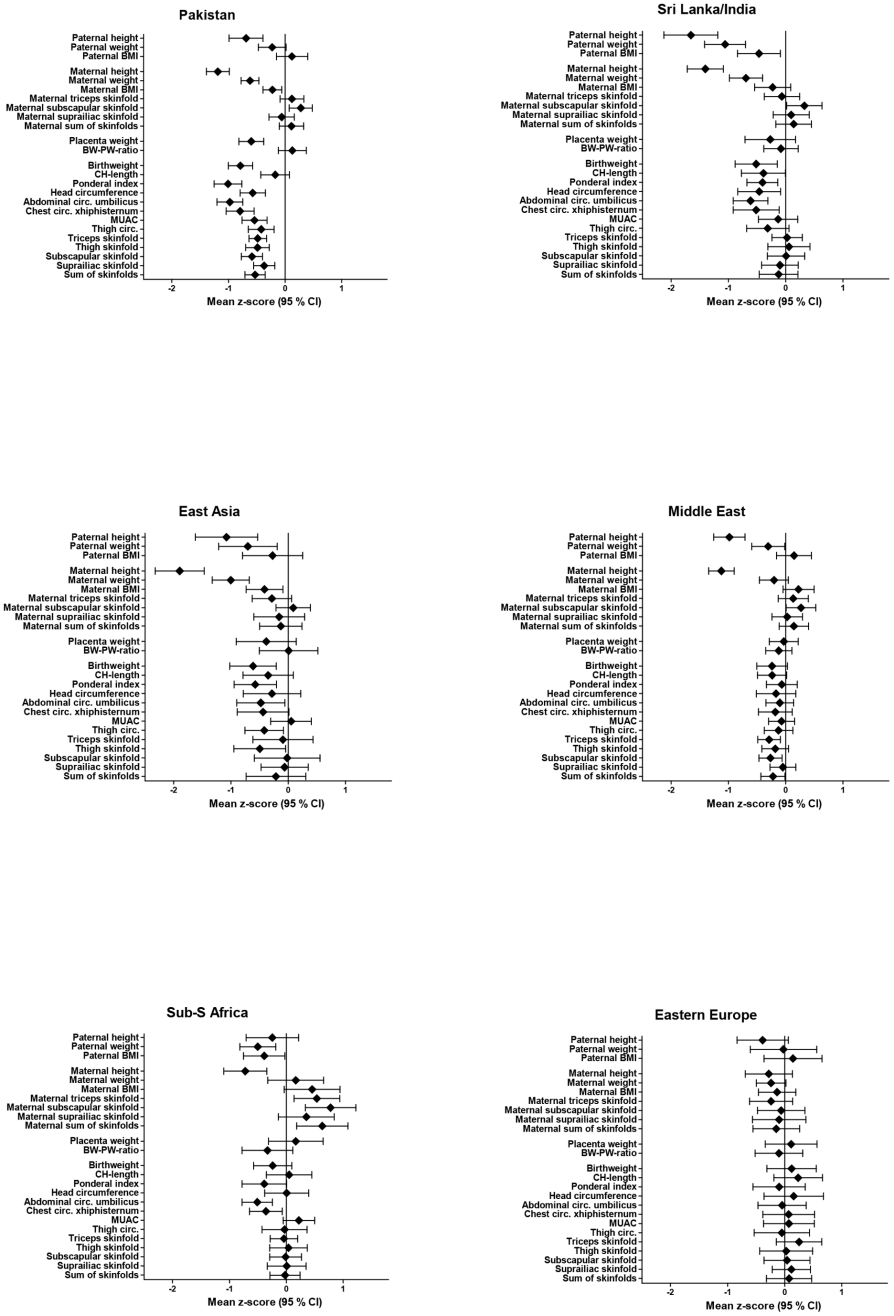
Mean z-scores (95% CI) of anthropometric measurements, for fathers, mothers, placentas and neonates in six ethnic minority groups (Pakistan, Sri Lanka/India, East Asia, Middle East, Sub-Sahara Africa and Eastern Europe), with Western Europeans as reference.

There were some variation between ethnic sub-groups in parental and neonatal characteristics (Table S1 and Table S2). Figure 4 presents mean z-scores for six of the ethnic minority sub-groups. The Pakistani group had the lowest BW, HC, AC, MUAC and skin folds. A significant heterogeneity across LAMIC ethnic minority sub-groups for BW (p = 0.039), AC (p<0.001) and ponderal index (p<0.001) was found; between the Pakistani and the Middle East group in BW (p = 0.023) and AC (p<0.001) and in ponderal index for the Pakistanis compared with both the Sri Lanka/India (p = 0.036) and the Middle East group (p<0.001). The especially thin neonatal phenotype in the Pakistani neonates, compared with the WEs, was also more evident after adjustments for parental factors.

Of 87 ethnic Pakistani women, 26 were born in Norway. When exploring potential differences between this group and those actually born in Pakistan, we found that ethnic Pakistani women born in Norway were significantly taller (mean z-score was 0.56 SD higher, p = 0.01). Other than that, maternal or neonatal anthropometry did not differ between the two groups. Hence, the neonatal phenotype, compared with WE neonates, were similar in ethnic Pakistani neonates, irrespective of country of birth of the mother.

## Discussion

To our knowledge, this is the first study to describe parental and neonatal anthropometry and body composition in detail in a multi-ethnic population, including ethnic groups of Asian, Middle East and African origin. LAMIC neonates had a “thin-fat phenotype”, as indicated by a smaller AC and lower ponderal index, but relatively preserved CH-length, HC and skin folds, compared with their WE counterparts. This pattern was particularly evident in the ethnic Pakistanis. The LAMIC phenotype persisted, and was even more evident, after adjustments for parental factors, as they explained most of the ethnic differences in CH-length, HC and skin folds, whereas estimates for AC and ponderal index were relatively unaffected by adjustments.

Ethnic variation in neonatal body composition, apart from BW, is sparsely documented. Differences in neonatal body composition have been observed between neonates in European high income countries compared with neonates in Asian and African low income countries, with least variation in skeletal measurements, like HC and length [11]. The “thin-fat phenotype” was first described in Indian neonates born in a poor rural district, as they had substantially smaller AC (mean z-score: −2.38 (−2.48, −2.29)), but relatively preserved body fat, compared with babies born in UK [12], [13]. The same pattern was later confirmed in a small study of multigenerational Indian immigrants in the middle-income country Surinam, when comparing with the same UK reference cohort [28]. Body composition in 30 ethnic South Asian and 30 ethnic European infants, 6–12 weeks of age, were studied in a recent UK study [29]. South Asian infants had less fat-free mass than the WE infants, while fat mass was similar. Further, AC and HC were reduced, even when adjusting for CH-length. Our results are consistent with these findings and indicate that these differences are evident at birth. Another UK study recently showed that despite being markedly lighter, Pakistani infants had similar skin fold thicknesses and greater total fat, as indicated by cord leptin, for a given birth weight than White British infants, independent of whether the mother was UK-born or not [30].

In studies from the US non-hispanic black neonates were found to have less lean body mass, calculated from anthropometric measurements, compared with non-hispanic-white neonates, while fat mass did not differ [31]–[33]. Our observations were similar for offspring of Sub-Saharan African immigrant women in Norway, with a different migration history than non-hispanic black US-women. Detailed anthropometry, of neonates and their parents, have to our knowledge not been described previously in Asian, Middle East and African ethnic minorities living in a high income country.

The mean BWs in the various ethnic groups in our study are close to that of similar ethnic groups in other high-income countries [14]–[17], regardless of the generational status of the mother [34], and higher than reported in the South Asian studies. As expected, the differences in neonatal body composition between ethnic WE and South Asian neonates were less extreme in our study compared with Indian neonates. If a trend towards a WE phenotype is to be regarded beneficial, this may reflect an improved health in women after migration to a high-income country. On the other hand, it could also reflect that women who migrate have a better health and socioeconomic background that those who do not. The observed difference in neonatal body composition between neonates with WE and LAMIC origin, and the heterogeneity within our LAMIC group, could partly be caused by genetic variation. However, countries categorized as WE or LAMIC, and countries within the LAMIC category, differ markedly with respect to geography and culture as well as economical resources and development. Through different mechanisms, including epigenetic processes, it is postulated that the mother limits fetal growth to be appropriate to her stature, parity and pre-pregnant condition, reflecting her past environment and her current nutritional status [35]. The fetus will thereby develop a phenotype appropriate to the environment in which it is likely to live [35]. Thus, epigenetic mechanisms may also contribute to the observed ethnic variation.

A mismatch between “expected” and actual postnatal environment may increase the risk of adult disease [36]. Several large ethnic minority groups from LAMIC in Norway have higher diabetes risk at lesser degree of adiposity than Norwegians [9]. Both an increased fat mass and a reduction in lean mass at birth are postulated to represent an increased risk for later type 2 diabetes [4], [37]. The “thin-fat phenotype” previously described in South Asians, represented by small abdominal viscera and low muscle mass, but preserved body fat, is regarded as an important contributing factor to the high incidence of type 2 diabetes in this region. In particular if followed by an obesogenic post-natal environment, this phenotype may predispose to an insulin-resistant state [8]. Hence, if the smaller ACs in LAMIC neonates reflects impaired growth of internal organs, such as liver and pancreas, it may have long term consequences for glucose homeostasis also in European ethnic minority populations [38].

In a study comparing cohorts from high vs. low and middle income countries, differences in maternal height and BMI explained most of the variation in neonatal BW between geographical (world) regions [39]. Our results suggest that growth of lean mass is less affected by differences in key parental factors, than growth of head, length and fat mass. Thus, the thin-fat phenotype in ethnic minority neonates and the increased susceptibility for type 2 diabetes and cardiovascular disease may persist over at least some generations, despite improved social conditions and larger parental size.

Strengths of our study include the population-based cohort design, a high attendance rate and minor loss to follow-up in the total cohort. We have extensive data collected prospectively during pregnancy and at birth, closely monitored, to ensure high quality of the data. Z-scores obtained from national data indicate that our WE group is representative for the ethnic Norwegian population and that the eligible babies without study specific measurements were missed at random due to logistic reasons. Inter- and intra-rater variability were comparable with similar high quality studies [40]. However, paternal height and weight were self-reported, and the number of fathers filling in the forms was lower in some ethnic groups. This caused loss of power and could potentially cause selection bias. Paternal data were therefore only analysed in a sub-sample.

“Body composition” is usually presented as percentage of body fat (% BF) and percentage of lean body mass. Our anthropometric measurements are proxy measures for body composition, although regarded appropriate in large scale studies. It is also important to be aware that skin fold measures mainly reflect superficial subcutaneous fat. Other studies have used formulas to predict BF from anthropometric measures (i.e. BW, CH-length and suprailiac skin fold) [41]. To calculate a valid fat percentage for an individual or a group based on these formulas, the distribution of superficial and deep subcutaneous and intra-abdominal fat, as well as body geometry, should be similar to the reference population [42]. Several studies have indicated that these assumptions are not necessarily met for all ethnic groups, in particular South Asians [11], [12], or for neonates exposed to intra-uterine growth retardation [43]. Hence, we did not use these formulas, as they may introduce systematic bias.

The LAMIC sample is a broad category, representing the ethnic minority groups living in this area. However, these groups represent large and growing minorities in most high-income countries today. Although some of the ethnic minority sub-groups were too small to detect statistically significant differences between them, the relatively large sample size made it possible to split Asians into relevant sub-categories. As we did not identify other studies describing neonatal body composition in a similar multi-ethnic setting, our study should add new knowledge to the discussion of ethnic differences in perinatal, and potentially also adult health outcomes.

### Conclusion

The thin-fat neonatal phenotype, observed in some low and middle income countries was also found in ethnic minority neonates in a multi-ethnic population in Norway. This phenotype, which may predispose to adult type 2 diabetes, was not explained by parental size, parity, age or educational level, and might originate from genetic, trans-generational epigenetic or environmental factors acting over the entire parental life-course. More knowledge about these relationships is necessary for developing appropriate interventions to prevent obesity and later adult disease in this population.

## Supporting Information

Table S1 **Detailed parental characteristics.**
(DOCX)

Table S2 **Detailed neonatal characteristics and anthropometric measurements.** ^h^ Includes placenta, cord and membranes.
(DOCX)
