# Supplementary material for: Ethnic Differences in Neonatal Body Composition in a Multi-Ethnic Population and the Impact of Parental Factors: A Population-Based Cohort Study
Source: PLoS One. 2013 Aug 29;8(8):e73058. doi: 10.1371/journal.pone.0073058 (PMC3756946; doi:10.1371/journal.pone.0073058)
Supplement: Table S1 — Detailed parental characteristics. (DOCX) [file pone.0073058.s001.docx]

**Table S1: Detailed parental characteristics.** Data presented as mean (sd) or n (%), by mother’s ethnic sub-groups.

|  | | **Western Europe^a^** | **Eastern Europe^b^** | **Pakistan^c^** | **Sri Lanka/**  **India^d^** | **East Asia^e^** | **Middle East^f^** | **Sub-Sahara Africa^g^** | **S-C-America^h^** |
| --- | --- | --- | --- | --- | --- | --- | --- | --- | --- |
| **Mothers (n=537)** | | n=229 | n=26 | n=87 | n=49 | n=27 | n=75 | n=36 | n=8 |
| Age at inclusion, years | | 30.7 (4.5) | 29.0 (4.0) | 28.4 (4.7) | 28.8 (4.5) | 30.7 (4.2) | 29.6 (5.6) | 28.6 (4.8) | 28.9 (6.7) |
| Primipara (%) | | 118 (52) | 15 (58) | 29 (33) | 25 (51) | 11 (41) | 24 (32) | 15 (42) | 1 (13) |
| Educational level | |  |  |  |  |  |  |  |  |
|  | Primary or less (< 10 years) (%) | 7 (3) | 4 (15) | 15 (17) | 10 (20) | 6 (22) | 26 (34) | 20 (56) | 0 (0) |
|  | High school (10-12 years) (%) | 76 (33) | 7 (27) | 43 (50) | 26 (53) | 11 (41) | 36 (48) | 12 (33) | 4 (50) |
|  | College/university education (%) | 145 (64) | 14 (53) | 29 (33) | 12 (25) | 10 (37) | 12 (16) | 4 (11) | 4 (50) |
| Norwegian born ( %) | | 209 (91) | 0 | 26 (30) | 0 | 0 | 4 (5) | 1 (3) | 0 (0) |
| Gestational weeks at inclusion. | | 14.2 (2.2) | 14.7 (3.7) | 15.9 (4.2) | 15.3 (3.6) | 15.7 (3.9) | 15.0 (3.4) | 17.3 (4.9) | 15.9 (3.8) |
| Anthropometrics | |  |  |  |  |  |  |  |  |
|  | Height , cm | 167.5 (5.7) | 165.9 (5.9) | 160.7 (5.4) | 159.5 (6.3) | 156.7 (6.1) | 161.1 (5.5) | 163.4 (6.4) | 162.5 (7.8) |
|  | Weight, kg | 70.7 (13.2) | 67.6 (8.4) | 62.6 (9.8) | 61.6 (13.3) | 57.5 (10.8) | 68.0 (14.5) | 72.9 (19.1) | 78.8 (22.2) |
|  | BMI, kg/m^2^ | 25.2 (4.5) | 24.6 (3.5) | 24.2 (3.5) | 24.2 (5.0) | 23.3 (3.7) | 26.2 (5.3) | 27.3 (6.6) | 29.5 (6.2) |
|  | Triceps skin fold, mm | 24.1 (7.1) | 22.4 (6.3) | 25.0 (6.6) | 23.6 (7.3) | 22.1 (6.2) | 25.0 (7.8) | 25.0 (7.8) | 27.9 (7.6) |
|  | Subscapular skin fold, mm | 19.4 (7.6) | 18.9 (7.4) | 21.5 (6.9) | 21.8 (7.8) | 20.0 (5.8) | 21.4 (8.3) | 25.3 (9.0) | 22.8 (7.0) |
|  | Suprailiac skin fold, mm | 26.8 (7.3) | 26.1 (8.1) | 26.4 (7.2) | 27.5 (7.7) | 25.7 (8.2) | 27.0 (8.2) | 29.4 (9.4) | 27.0 (8.5) |
|  | Sum of skin folds, mm | 70.3 (19.5) | 67.4 (18.9) | 72.5 (18.6) | 73.0 (20.2) | 67.8 (18.2) | 73.1 (21.1) | 88.0 (22.2) | 74.7 (20.8) |
| **Fathers with complete data (n=414)** | | n=209 (91) | n=16 (62) | 56 (64) | 31 (63) | 23 (85) | 54 (72) | 19 (53) | 7 (88) |
| Ethnicity same as mother (%) | | 190 (92) | 8 (50) | 53 (95) | 31 (100) | 13 (56) | 53 (98) | 19 (100) | 2 (29) |
| Height, cm | | 181.1 (6.3) | 178.6 (5.3) | 176.8 (7.2) | 170.6 (8.1) | 174.3 (8.0) | 175.0 (6.4) | 179.5 (6.1) | 172.7 (11.2) |
| BMI, kg/m^2^ | | 26.5 (3.8) | 27.1 (3.6) | 27.0 (3.9) | 24.8 (3.9) | 25.5 (4.6) | 27.1 (4.2) | 27.1 (2.9) | 25.0 (1.7) |

^a^Western Europe (n=229, 7 from other Scandinavian countries than Norway, 5 with other Western-European background (3 born in North America))

^b^ Eastern Europe (n=26, Former Soviet Union: 23 %, other Former Easter Bloc- countries: 38 % and Former Yugoslavia: 38 %)

^c^ Pakistan (1 from Bangladesh)

^d^ Sri Lanka/India (n=49, 12 % from India, 88 % from Sri Lankan, almost exclusively Tamils)

^e^ East Asia (n= 27, largest groups: Vietnam: 44 % and Philippines: 26 %)

^f^ Middle East (n= 75, largest groups Iraq: 28 %, Morocco: 20 %, Turkey: 20 % and Afghanistan: 13 %)

^g^ Sub-Sahara Africa (n=36, largest group Somalia 64 %)

^h^ South-/Central-America and Caribbean.
