# Supplementary material for: Ethnic Differences in Neonatal Body Composition in a Multi-Ethnic Population and the Impact of Parental Factors: A Population-Based Cohort Study
Source: PLoS One. 2013 Aug 29;8(8):e73058. doi: 10.1371/journal.pone.0073058 (PMC3756946; doi:10.1371/journal.pone.0073058)
Supplement: Table S2 — Detailed neonatal characteristics and anthropometric measurements. h Includes placenta, cord and membranes. (DOCX) [file pone.0073058.s002.docx]

**Table S2**: **Detailed neonatal characteristics and anthropometric measurements.** Data presented as mean (sd) or n (%) by ethnic sub-group.

|  |  | **Western**  **Europe** | **Eastern**  **Europe** | **Pakistan** | **Sri Lanka/**  **India** | **East**  **Asia** | **Middle East** | **Sub-Sahara Africa** | **S/C-America** |
| --- | --- | --- | --- | --- | --- | --- | --- | --- | --- |
|  |  | n=229 | n=26 | n=87 | n=49 | n=27 | n=75 | n=36 | n=8 |
|  | Gestational age, days ^a^ | 283 (9) | 283 (8) | 280 (9) | 279 (8) | 281 (8) | 277 (9) | 282 (10) | 276 (10) |
|  | Gender, boy (%) | 119 (52) | 12 (46) | 42 (48) | 29 (59) | 16 (59) | 30 (40) | 14 (39) | 6 (75) |
|  | Placenta weight, g^b^ | 696 (142) | 727 (166) | 619 (128) | 652 (168) | 650 (174) | 677 (125) | 710 (172) | 654 (156) |
|  | Birth weight, g | 3600 (467) | 3676 (509) | 3233 (441) | 3314 (490) | 3338 (467) | 3383 (512) | 3460 (458) | 3229 (411) |
|  | CH-length, cm^c^ | 50.0 (1.9) | 50.6 (1.9) | 49.6 (2.0) | 49.2 (1.8) | 49.5 (2.0) | 49.1 (1.9) | 50.0 (1.7) | 49.7 (2.1) |
|  | Ponderal index , kg/m^3^ | 28.7 (2.4) | 28.2 (2.1) | 26.4 (2.4) | 27.8 (2.0) | 27.5 (2.0) | 28.7 (2.5) | 27.6 (2.6) | 26.3 (2.4) |
| Circumferences | |  |  |  |  |  |  |  |  |
|  | Head, cm | 35.1 (1.3) | 35.3 (1.3) | 34.4 (1.2) | 34.5 (1.5) | 34.8 (1.6) | 34.6 (1.6) | 35.0 (1.3) | 34.7 (1.3) |
|  | Abdomen (umbilicus), cm | 32.7 (2.2) | 32.8 (2.4) | 30.6 (2.0) | 31.2 (2.0) | 31.7 (2.4) | 32.1 (2.1) | 31.8 (1.8) | 30.9 (2.0) |
|  | Chest (xiphisternum), cm | 34.0 (1.8) | 34.3 (2.0) | 32.6 (1.7) | 32.9 (1.9) | 33.3 (1.9) | 33.3 (1.9) | 33.4 (1.6) | 32.9 (1.7) |
|  | Mid-upper-arm, cm | 11.5 (1.0) | 11.6 (1.1) | 11.0 (1.0) | 11.3 (1.0) | 11.6 (0.9) | 11.3 (1.0) | 11.7 (0.9) | 10.5 (0.7) |
|  | Thigh, cm | 16.2 (1.5) | 16.2 (1.8) | 15.5 (1.5) | 15.5 (1.6) | 15.5 (1.2) | 15.7 (1.5) | 16.6 (1.7) | 14.9 (1.2) |
| Skin folds | |  |  |  |  |  |  |  |  |
|  | Triceps, mm | 4.6 (1.1) | 4.8 (1.0) | 4.1 (0.8) | 4.5 (1.0) | 4.5 (1.3) | 4.3 (0.9) | 4.5 (0.9) | 4.0 (0.9) |
|  | Thigh, mm | 6.0 (1.3) | 6.1 (1.5) | 5.4 (1.2) | 6.0 (1.5) | 5.3 (1.6) | 5.8 (1.3) | 6.2 (1.2) | 4.7 (1.0) |
|  | Subscapular, mm | 4.5 (1.1) | 4.5 (1.0) | 3.9 (0.9) | 4.4 (1.1) | 4.6 (1.6) | 4.2 (0.9) | 4.5 (0.9) | 3.6 (0.5) |
|  | Suprailiac crest, mm | 3.6 (0.9) | 3.7 (0.8) | 3.2 (0.7) | 3.4 (0.9) | 3.5 (0.9) | 3.5 (0.8) | 3.6 (0.9) | 3.1 (0.7) |
|  | Sum skinfolds, mm | 18.7 (4.0) | 19.0 (3.9) | 16.5 (3.3) | 17.9 (4.4) | 17.9 (5.2) | 17.7 (3.5) | 18.7 (3.2) | 15.3 (2.7) |

^a^ Based on last menstrual period for all births in study sample, includes 37 with ultrasound-derived term

^b^ Includes placenta, cord and membranes.

^c^ Missing in 43 neonates (8 %), mostly due to intrauterine breech position, family history of hip-dysplasia or other circumstances restricting stretching of the baby.
